# Distributed representations of behaviorally-relevant object dimensions in the human visual system

**DOI:** 10.1101/2023.08.23.553812

**Published:** 2023-11-20

**Authors:** O. Contier, C.I. Baker, M.N. Hebart

**Affiliations:** 1 Vision and Computational Cognition Group, Max Planck Institute for Human Cognitive and Brain Sciences, Leipzig, Germany; 2 Max Planck School of Cognition, Leipzig, Germany; 3 Laboratory of Brain and Cognition, National Institute of Mental Health, National Institutes of Health, Bethesda MD, USA; 4 Department of Medicine, Justus Liebig University Giessen, Giessen, Germany

## Abstract

Object vision is commonly thought to involve a hierarchy of brain regions processing increasingly complex image features, with high-level visual cortex supporting object recognition and categorization. However, object vision supports diverse behavioral goals, suggesting basic limitations of this category-centric framework. To address these limitations, here we map a series of behaviorally-relevant dimensions derived from a large-scale analysis of human similarity judgments directly onto the brain. Our results reveal broadly distributed representations of behaviorally-relevant information, demonstrating selectivity to a wide variety of novel dimensions while capturing known selectivities for visual features and categories. Behaviorally-relevant dimensions were superior to categories at predicting brain responses, yielding mixed selectivity in much of visual cortex and sparse selectivity in category-selective clusters. This framework reconciles seemingly disparate findings regarding regional specialization, explaining category selectivity as a special case of sparse response profiles among representational dimensions, suggesting a behavior-centric view on visual processing in the human brain.

## Introduction

A central goal of visual neuroscience is to understand how the brain encodes and represents rich information about objects, allowing us to make sense of our visual world and act on it in meaningful ways. A widely studied and influential account posits that one central function of the visual system is to recognize objects by organizing them into distinct categories ^1–4^. According to this view, early visual cortex serves to analyze incoming visual information by representing basic visual features ^5^, which are then combined into more and more complex feature combinations, until higher-level visual regions in the occipitotemporal cortex and beyond support the recognition of object identity and category ^3^. In line with this view, a number of category-selective clusters have been identified in occipitotemporal cortex that respond selectively to specific object classes such as faces, scenes, body parts, tools, or text ^6–11^. The functional significance of these regions is underscored by studies demonstrating that object category and identity as well as performance in some behavioral tasks can be read out from activity in occipitotemporal cortex ^12–17^ and that lesions to these regions can lead to selective deficits in object recognition abilities ^18–22^.

Despite the importance of object categorization and identification as crucial goals of object vision, it has been argued that these functions alone are insufficient for capturing how our visual system allows us to make sense of the objects around us ^23^. A more comprehensive understanding of object vision should account for the rich meaning and behavioral relevance associated with individual objects beyond discrete labels. This requires incorporating the many visual and semantic properties of objects that underlie our ability to make sense of our visual environment, perform adaptive behaviors, and communicate about our visual world ^23–27^. Indeed, others have proposed that visual cortex is organized based on continuous dimensions reflecting more general object properties, such as animacy ^28–31^, real-world size ^29,32^, aspect ratio ^31,33^, or semantics ^34^. These and other continuous dimensions reflect behaviourally-relevant information that offers a more fine-grained account of object representations than discrete categorization and recognition alone. This dimensional view suggests a framework in which visual cortex is organized based on continuous topographic maps tuned to specific dimensions that extend beyond category-selective clusters. Under this framework, category-selective clusters may emerge from a more general organizing principle ^34–38^, reflecting cortical locations where these tuning maps encode feature combinations tied to specific object categories ^34,38,39^. Yet, while previously proposed dimensions have been shown to partially reflect activity patterns in category-selective clusters ^40–45^, they cannot account fully for the response profile and are largely inferior to category-selectivity in explaining the functional selectivity of human visual cortex for objects ^46,47^.

To move beyond the characterization of individual behavioral goals underlying both the discrete category-centric and the continuous dimensional view and to comprehensively map a broad spectrum of behaviorally-relevant representations, one powerful approach is to link object responses in visual cortex to judgments about the perceived similarity between objects ^48–51^. Indeed, perceived similarity serves as a common proxy of mental object representations underlying various behavioral goals, as the similarity relation between objects conveys much of the object knowledge and behavioral relevance across diverse perceptual and conceptual criteria ^52–56^. As such, perceived similarity is ideally suited for revealing behaviorally-relevant representational dimensions and how these dimensions are reflected in cortical patterns of brain activity.

To uncover the nature of behaviorally-relevant selectivity underlying similarity judgments in human visual cortex, in the present study we paired functional MRI responses to thousands of object images ^57^ with core representational dimensions derived from a dataset of millions of human similarity judgments. In contrast to much previous research that has focused on a small number of hypothesis-driven dimensions or that used small, selective image sets ^29,48–51,58–60^, we carried out a comprehensive characterization of cortical selectivity in response to 66 representational dimensions identified in a data-driven fashion for 1,854 objects ^52,61^.

Moving beyond the view that mental object representations derived from similarity judgments are primarily mirrored in high-level visual cortex ^48–50,57^, we demonstrate that representations underlying core object dimensions are reflected throughout the entire visual cortex. Our results reveal that cortical tuning to these dimensions captures the functional topography of visual cortex and mirrors stimulus selectivity throughout the visual hierarchy. In this multidimensional representation, category selectivity stands out as a special case of sparse selectivity to a set of core behaviorally-relevant representational object dimensions, while other parts of visual cortex reflect a more mixed selectivity. A direct model comparison revealed that continuous object dimensions provide a better model of brain responses than categories across the visual system, suggesting that dimension-related tuning maps offer more explanatory power than a category-centric framework. Together, our findings reveal the importance of behaviourally-relevant object dimensions for understanding the functional organization of the visual system and offer a broader, comprehensive view of object representations that bridges the gap between regional specialization and domain-general topography.

## Results

**Fig. 1.**
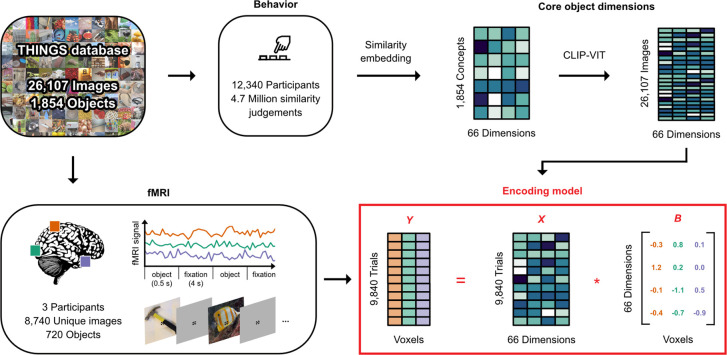
Overview: An fMRI encoding model of object dimensions underlying human similarity judgements. We linked core representational dimensions capturing the behavioral relevance of objects to spatially resolved neural responses to thousands of object images. For this, we used the THINGS-data collection ^57^ which includes fMRI and behavioral responses to objects from the THINGS object concept and image database ^66^. The behavioral data was used to train a computational model of core object dimensions underlying human similarity judgements to different object concepts. We extended this embedding to the level of individual object images based on the computer vision model CLIP-VIT ^64^. The fMRI data comprises three participants who each saw 8,740 unique object images. We used an encoding model of the object dimension embedding to predict fMRI responses to each image in each voxel. The estimated encoding model weights reflect the tuning of each voxel to each object dimension. *X*, *B*, and *Y* denote the design matrix, regression weights, and outcome of the encoding mode, respectively.

We first aimed at mapping core representational object dimensions to patterns of brain activity associated with visually-perceived objects. To model the neural representation of objects while accounting for their large visual and semantic variability ^62,63^, we used the THINGS-data collection ^57^, which includes densely sampled fMRI data for thousands of naturalistic object images from 720 semantically diverse objects, as well as 4.7 million behavioral similarity judgments of these objects (Fig. 1).

As core object dimensions, we used a recent similarity embedding of behaviorally-relevant object dimensions, which underlie the perceived similarity of 1,854 object concepts ^52,57^. In this embedding, each object image is characterized by 66 dimensions derived from the human similarity judgments in an odd-one-out task. We chose this embedding for several reasons: First, it provides highly reproducible dimensions that together are sufficient for capturing single trial object similarity judgments close to the noise ceiling. Second, the use of an odd-one-out task supports the identification of the minimal information required to distinguish between different objects, and as such is sensitive not only to conceptual information, such as high-level category (e.g., “is an animal”), but also to key visual-perceptual distinctions (e.g., “is round”). Indeed, the object dimensions capture external behavior such as categorization and typicality judgements, underscoring their behavioral relevance as a model of neural responses to objects ^52^. Third, the object dimensions are easily interpretable, thus simplifying the interpretation of neural activity patterns in relation to individual dimensions.

The fMRI dataset covers 8,740 unique images from 720 categories presented to three participants (2 female) over the course of 12 sessions ^57^. Given that the behavioral similarity embedding was trained only on one image per each of the 1,854 THINGS categories, these dimensions may only partially capture the visual richness of the entire image set, which may affect the potential for predicting image-wise brain responses. To address this challenge, we fine-tuned the artificial neural network model CLIP-VIT ^64^ to directly predict object dimensions for the 8,740 images in our fMRI dataset. This approach led to highly accurate cross-validated predictions of object similarity ^65^ and consistent improvements in BOLD signal predictions for all 66 dimensions (Suppl. Fig. 1).

### Core object dimensions are reflected in widespread fMRI activity patterns throughout the human visual system

To test how these dimensions were expressed in voxel-wise brain responses, we fit an fMRI encoding model which predicts spatially resolved brain responses based on a weighted sum of these object dimensions. This allowed us to map out the contribution of the dimensions to the measured signal and thus link interpretable behaviorally-relevant dimensions to patterns of brain activity.

**Fig. 2.**
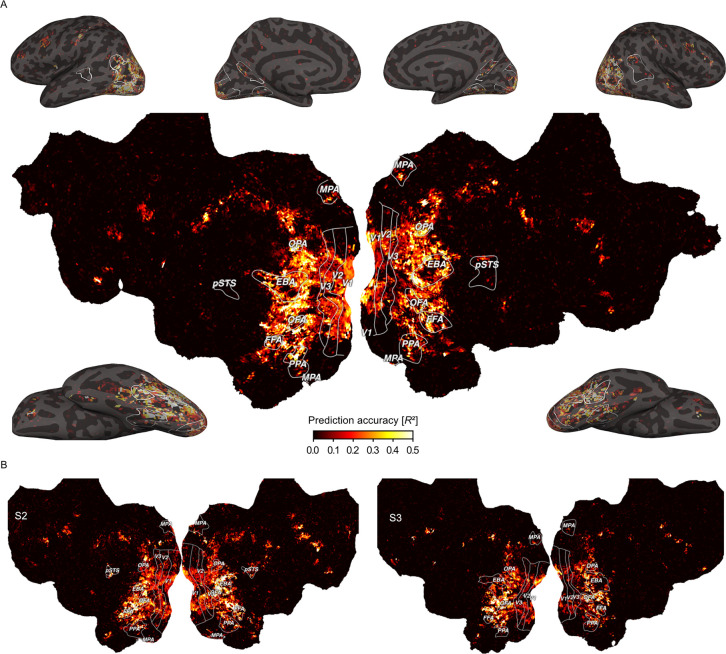
Prediction accuracy of the fMRI voxel-wise encoding model based on 66 core object dimensions. Colors indicate the proportion of explained variance (noise ceiling corrected R^2^) of held-out data in a 12-fold between-session cross-validation. White outlines indicate regions of interests defined in separate localizer experiments: FFA: Fusiform face area; OFA: Occipital face area; pSTS: Posterior superior temporal sulcus; EBA: Extrastriate body area; PPA: Parahippocampal place area; OPA: Occipitoparietal place area; MPA: Medial place area; V1-V3: Primary to tertiary visual cortex. A. Prediction accuracy for one example subject (S1) visualized on a cortical flat map (center) and inflated views of the cortical surface (corners). B. Results for the other two subjects visualized on cortical flat maps.

Across all 66 object dimensions, our results revealed a widely distributed cortical representation of these dimensions that spans much of visual cortex and beyond (Fig. 2). The spatial extent of these effects was consistent across all three subjects, underscoring the generality of these findings. Prediction accuracies peaked not only in lateral occipital and posterior ventral temporal regions, but also reached significant values in early visual, dorsal visual, and frontal regions (Suppl. Fig. 2). In contrast to previous work based on representational similarity analysis that found information about perceived similarity to be confined primarily to higher-level visual cortex ^49–51,57^, our dimension-based approach revealed that behaviorally-relevant information about objects is much more distributed throughout the visual processing hierarchy, including the earliest cortical processing stages.

### Behaviorally-relevant object dimensions reflect the functional topography of the human visual system

Having identified where information about perceived similarity is encoded, we next explored the spatial layout of each individual dimension underlying this representation. By using a voxel-encoding model of interpretable object dimensions, it is possible to inspect the cortical distribution of the weights of each regressor separately and interpret them in a meaningful fashion. This has two benefits. First, it allows us to probe to what degree behaviorally-relevant dimensions alone can capture the known topography of visual cortex. Second, it allows us to identify novel topographic patterns across visual cortex. This provides important insights into how the topography of visual cortex reflects object information relevant to behavior and how functionally specialized regions are situated in this cortical landscape.

**Fig. 3.**
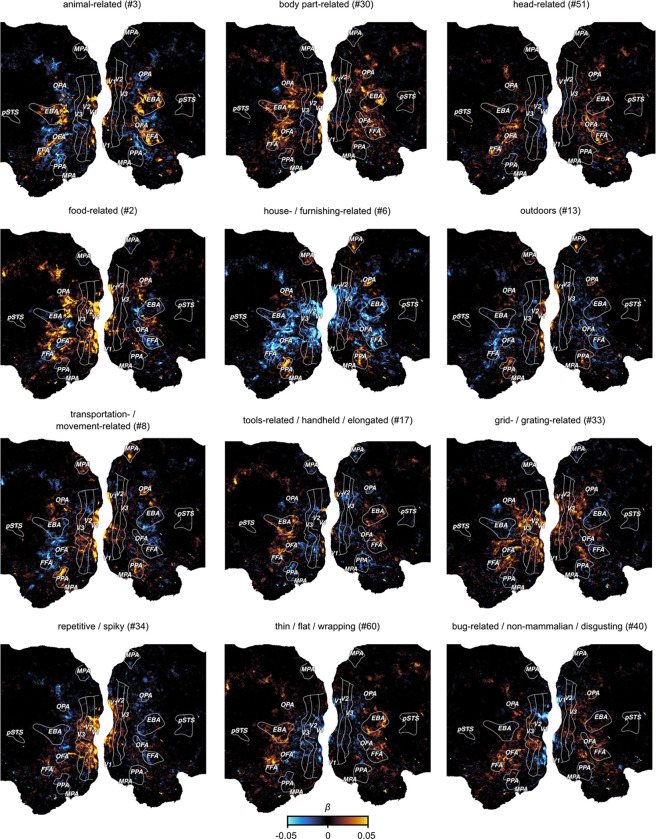
Functional tuning maps to individual object dimensions. The figure shows example maps for 12 out of the 66 dimensions for Subject S1. Each panel shows the encoding model weights for one object dimension projected onto the flattened cortical surface. Numbers in the subtitles show the dimension number in the embedding.

Visualizing the voxel-wise regression weights for each object dimension on the cortical surface (Fig. 3) revealed a clear correspondence between numerous dimensions and characteristic, known topographic patterns of the visual system. For example, the “animal-related” dimension mirrors the well established spoke-like tuning gradient for animate versus inanimate objects ^29^, while dimensions like “head-related” and “body-part related” differentiate the regional selectivity for faces and body parts in the fusiform face area (FFA), occipital face area (OFA), and extrastriate body area (EBA), respectively ^6,7,67^. Likewise, the implicit inclusion of natural scenes as object backgrounds revealed scene content-related dimensions (e.g. “house-/furnishing-related”, “transportation-/movement-related”, and “outdoors”), which were found to be associated with scene-selective brain regions such as parahippocampal place area (PPA), medial place area (MPA), and occipital place area (OPA) ^8,68–72^.^8,68–71^Our approach also independently identified a “food-related” dimension in areas adjacent to the fusiform gyrus, in line with recently reported clusters responding selectively to food stimuli ^73–75^. A dimension related to tools (“tool-related/handheld/elongated”) also matched expected activation patterns in middle temporal gyrus ^11,76,77^. Further, dimensions related to low- to mid-level visual features (e.g. “grid/grating-related”, “repetitive/spiky”) reflected responses primarily in early visual cortex.

**Extended Data Figure 1.**
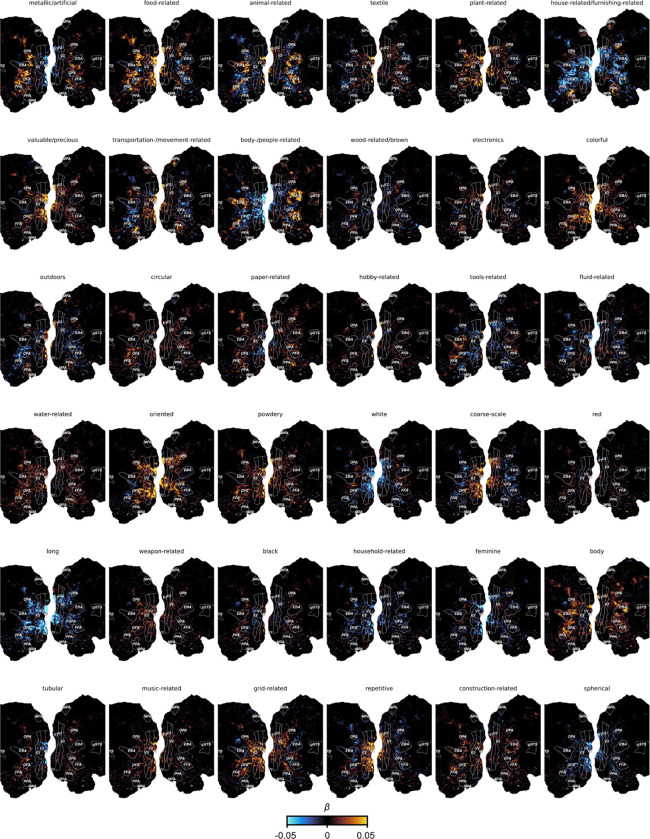
Dimension tuning maps 1–36 for Subject 1. Colors indicate regression weights for each dimension predictor from the parametric modulation encoding model. Labels indicate regions of interest on the cortex: V1-V3: primary - tertiary visual cortex, OFA: occipital face area, FFA: fusiform face area, EBA: extrastriate body area, PPA: parahippocampal place area, MPA: medial place area, OPA: occipital place area.

**Extended Data Figure 2.**
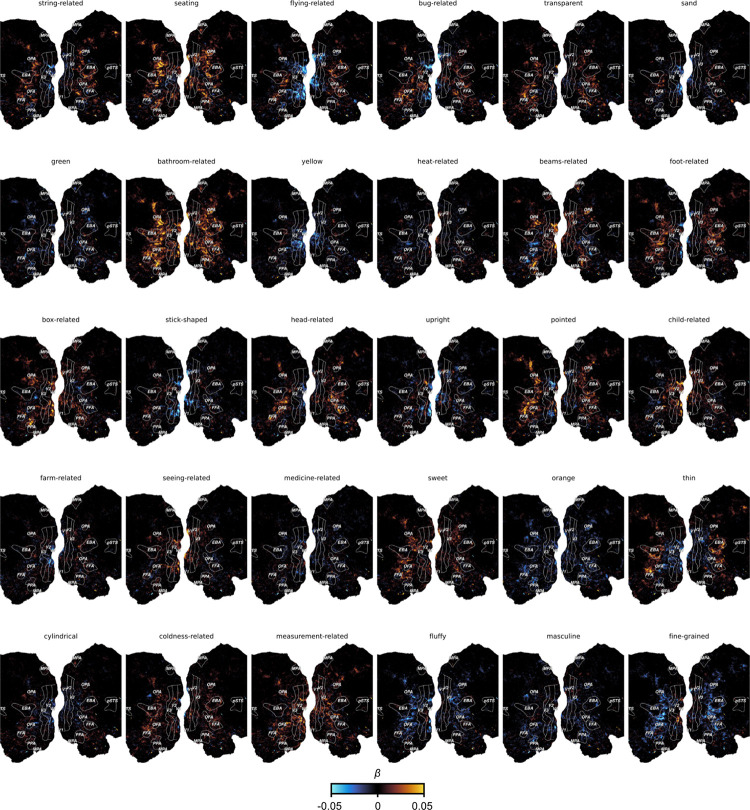
Dimension tuning maps 37–66 for Subject 1. Colors indicate regression weights for each dimension predictor from the parametric modulation encoding model.

**Extended Data Figure 3.**
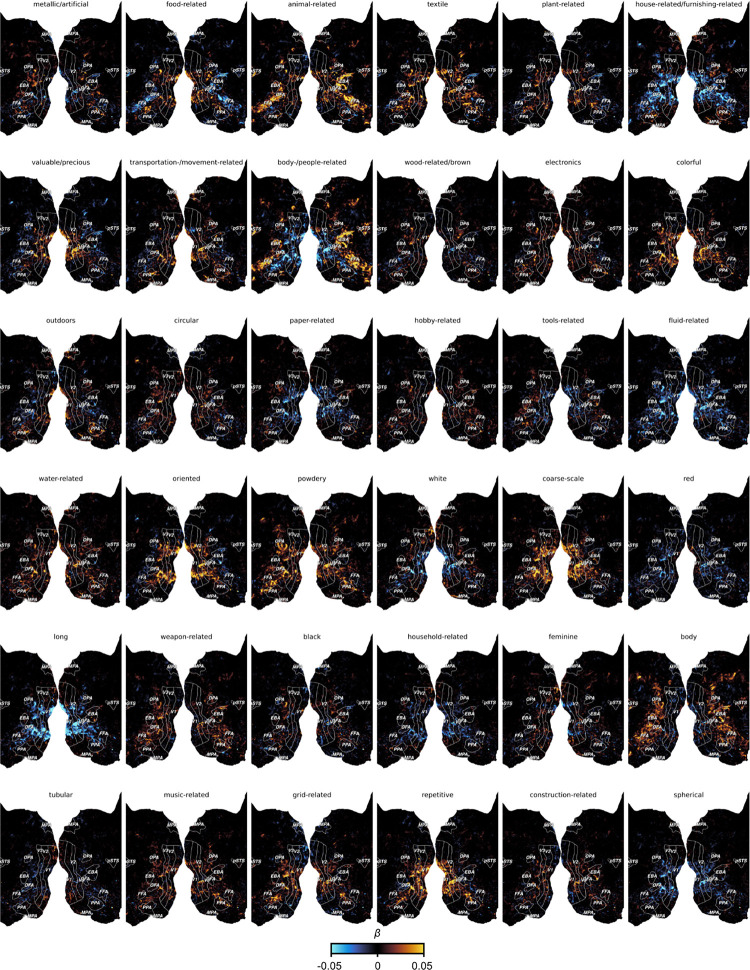
Dimension tuning maps 1–36 for Subject 2. Colors indicate regression weights for each dimension predictor from the parametric modulation encoding model.

**Extended Data Figure 4.**
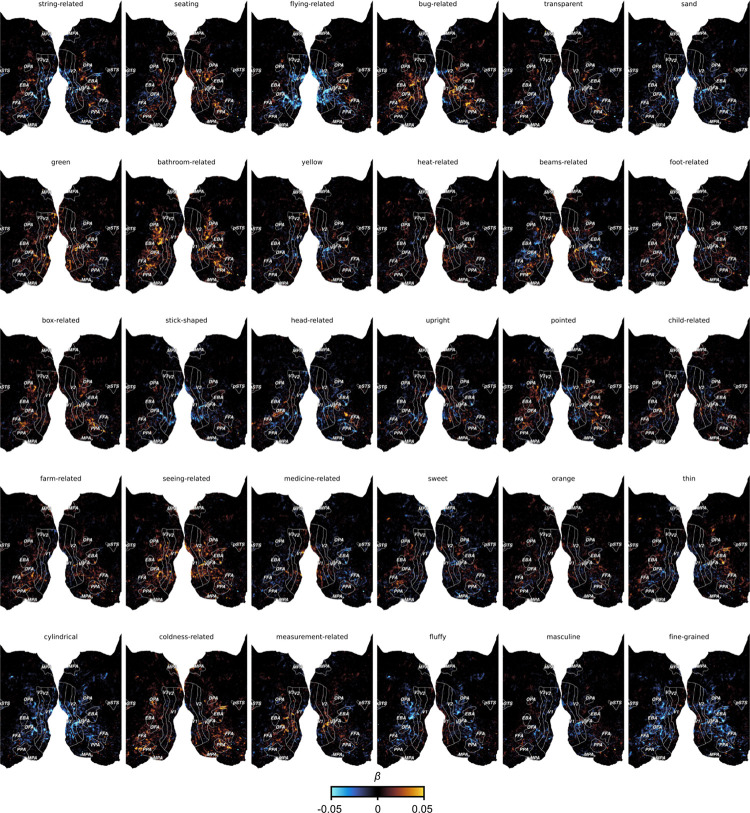
Dimension tuning maps 37–66 for Subject 2. Colors indicate regression weights for each dimension predictor from the parametric modulation encoding model.

**Extended Data Figure 5.**
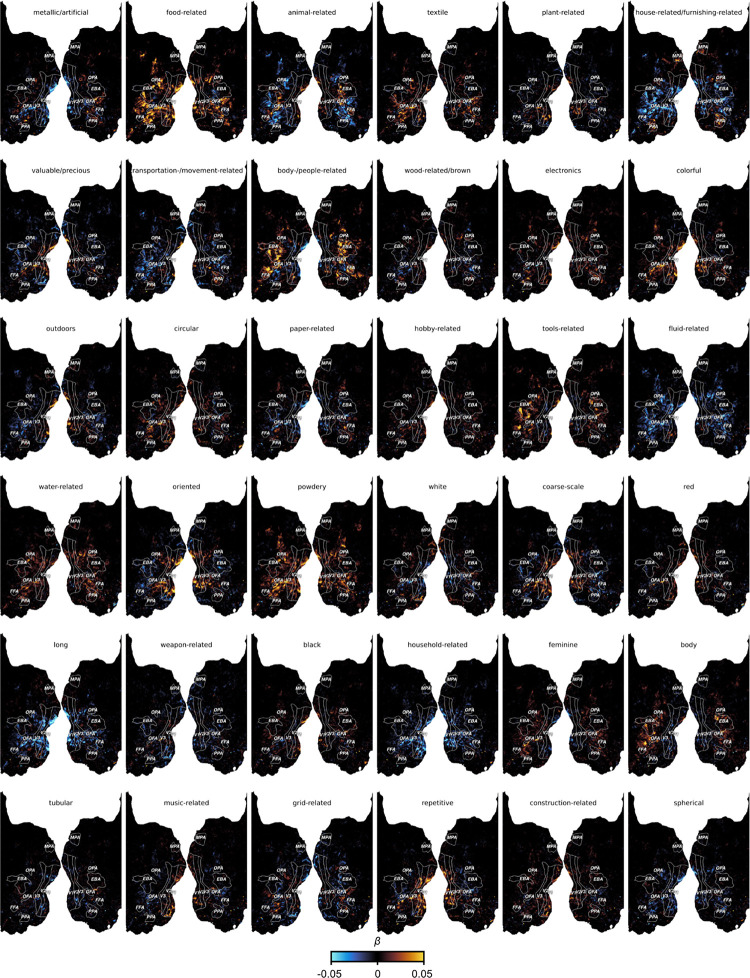
Dimension tuning maps 1–36 for Subject 3. Colors indicate regression weights for each dimension predictor from the parametric modulation encoding model.

**Extended Data Figure 6.**
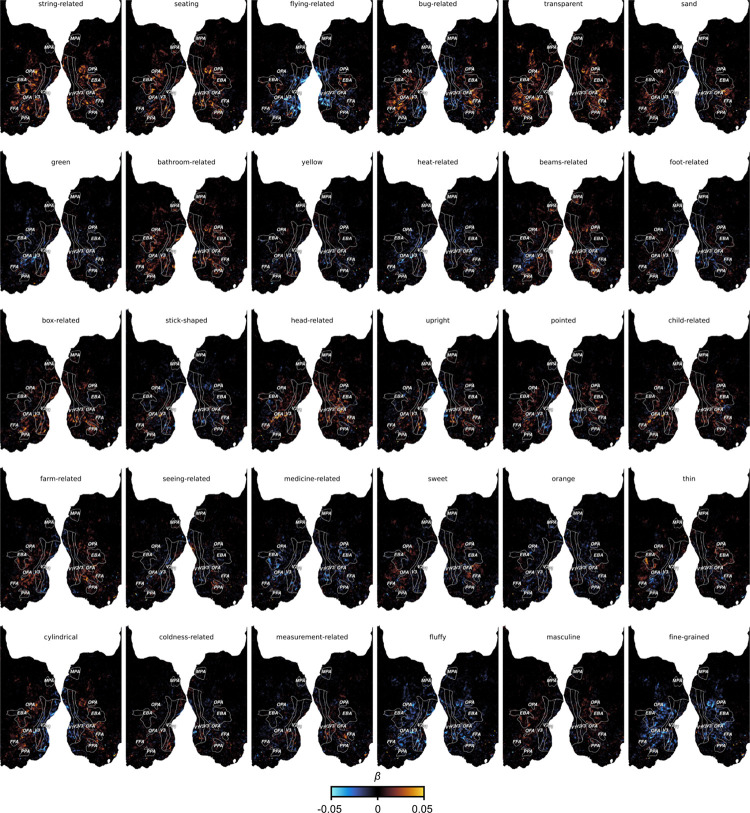
Dimension tuning maps 37–66 for Subject 3. Colors indicate regression weights for each dimension predictor from the parametric modulation encoding model.

Beyond these established topographies, the results also revealed numerous additional topographic patterns. For example, one dimension reflected small, non-mammalian animals (“bug-related / non-mammalian / disgusting”) that was clearly distinct from the “animal-related” dimension by lacking responses in face and body selective regions. Another dimension reflected a widely distributed pattern in response to thin, flat objects (“thin / flat / wrapping”). Thus, our approach allowed for the identification of candidate functional selectivities in visual cortex that might have gone undetected with more traditional approaches based on proposed categories or features ^47,73^. Importantly, the functional topographies of most object dimensions were also found to be highly consistent across the three subjects, suggesting that they may reflect general organizing principles rather than idiosyncratic effects (Extended Data Fig. 1–6).

Together, our results uncover cortical maps of object dimensions underlying the perceived similarity between objects. These maps span extensive portions of the visual cortex, capturing topographic characteristics such as tuning gradients of object animacy, lower-level visual feature tuning in early visual cortex, and category-selective, higher-level regions while uncovering new candidate selectivities. Thus, these findings support an organizing principle where multiple, superimposing cortical tuning maps for core object properties collectively represent behaviorally-relevant information of objects.

### Cortical tuning to behaviorally-relevant object dimensions explains regional functional selectivity

Having delineated the multidimensional topographic maps across visual cortex, we next honed in on individual brain regions to determine their functional selectivity as defined by their response tuning across these behaviorally-relevant dimensions. To this end, we developed a high-throughput method to identify object images representative for specific brain regions. Specifically, we first determined a functional tuning profile across dimensions for each region of interest based on the region’s mean encoding model weights. Next, we identified images whose behavioral dimension profile best matched the functional tuning profile of the brain region. To this end, we used all 26,107 object images in the THINGS database ^66^, most of which were unseen by participants, and assessed the cosine similarity between the dimension profiles of brain regions and images. This enabled us to rank over 26,000 images based on their similarity to a given brain region’s functional tuning profile.

**Fig. 4.**
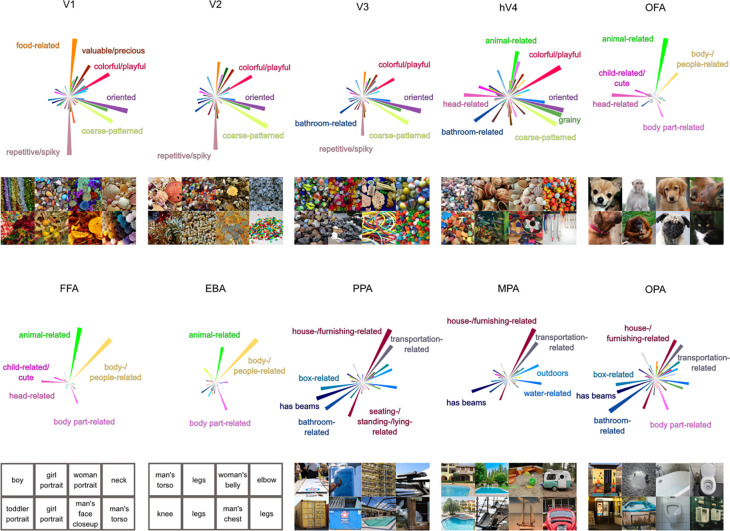
Regional tuning profiles across 66 object dimensions and representative images for selectivity of each region of interest in visual cortex. Rose plots indicate the magnitude of tuning for each object dimension in a given visual brain region. Image panels show 8 images with the most similar model representation to the regional tuning profile. For copyright reasons, all original images have been replaced with visually similar images. For identity protection reasons, images containing human faces and body parts have replaced with verbal descriptions. Original images are available upon request.

Despite having been fitted solely on the 66-dimensional similarity embedding, our approach successfully identified diverse functional selectivities of visual brain regions (Fig. 4). For instance, the most representative images for early visual regions (V1, V2, V3) contained fine-scale, colorful, and repeating visual features, consistent with known representations of oriented edges and color in these areas ^78,79^. These patterns appeared more fine-grained in earlier (V1 or V2) compared to later retinotopic regions (hV4), potentially reflecting increased receptive field size along the retinotopic hierarchy ^80–82^. A similar finding is reflected in dimension selectivity profiles (Fig. 4), revealing increased color selectivity in hV4 compared to early retinotopic regions V1-V3 while yielding reductions in the “repetitive/spiky” dimension. Notably, tuning profiles in category-selective regions aligned with images of expected object categories: faces in face-selective regions (FFA, OFA), body parts in body-part selective regions (EBA), and scenes in scene-selective regions (PPA, OPA, MPA). Closer inspection of the tuning profiles revealed differences between regions that respond to the same basic object category, such as a stronger response to the “body-part related” dimension in OPA but not in other place-selective regions. Also, selectivity to faces (FFA, OFA) vs. body parts (EBA) appeared to be driven by the response magnitude to the “head-related” dimension, while tuning to the remaining dimensions was highly similar across these regions. Together, these findings demonstrate that the 66 object dimensions derived from behavior capture the selectivity across the visual processing hierarchy, highlighting the explanatory power of the dimensional framework for characterizing the functional architecture of the visual system.

### Category-selective brain regions are sparsely tuned to behaviorally-relevant object dimensions

Given that dimensional tuning profiles effectively captured the selectivity of diverse visual regions, we asked what factors distinguish category-selective visual brain regions from non-category-selective regions in this dimensional framework. We reasoned that category-selectivity reflects a sparsely tuned representation, where activity in category-selective regions is driven by only a few dimensions, while non-category-selective regions reflect a more mixed selectivity, with activity related to a larger number of dimensions. In this way, functionally specialized, category-selective regions might stand-out as an extreme case of multidimensional tuning. As a consequence, this would also make it easier to identify category-selective regions due to their sparser selectivity.

**Fig. 5.**
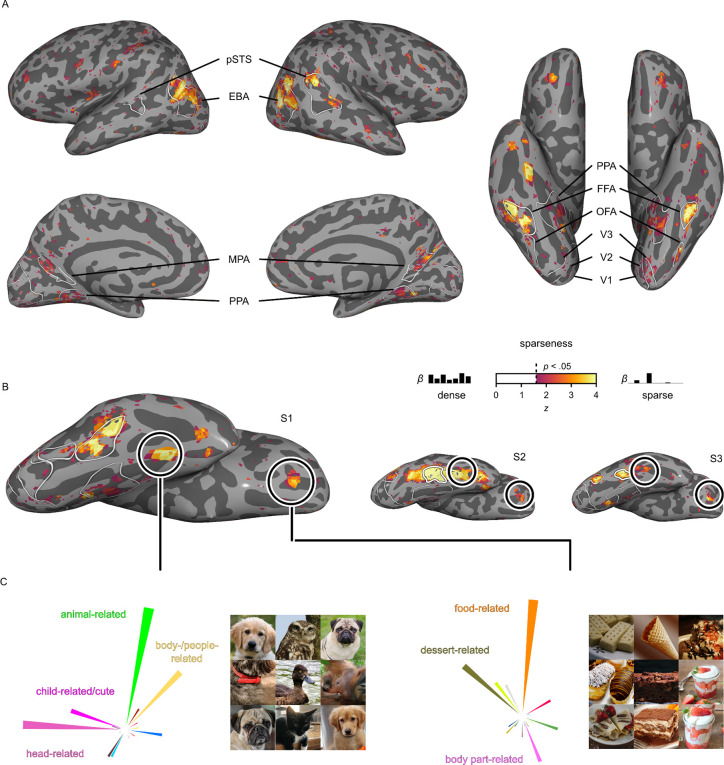
Representational sparseness of behaviorally-relevant object dimensions in object category selective brain regions. A. Inflated cortical surfaces for Subject 1 showing the sparseness over the encoding model weights in each voxel. Colors indicate z-values of sparseness compared to a noise pool of voxels. Statistical maps are thresholded at *p* < 0.05. B. Ventral view of the right hemisphere for all three subjects. Round outlines illustrate the location of two explorative, sparsely tuned regions of interest: One in the fusiform gyrus and one in orbitofrontal cortex. C. Functional selectivity of these explorative regions of interest demonstrated by their multidimensional tuning profiles and most representative object images. For copyright reasons, all original images have been replaced with visually similar images. Original images are available upon request.

To quantify this, we estimated a measure of sparseness over the encoding model weights in each voxel. Large sparseness indicates regions that are selective to very few dimensions, while lower sparseness indicates a dense representation in regions that respond broadly to diverse dimensions. Our results (Fig. 5A) indeed revealed sparser tuning in category-selective regions compared to other parts of the visual system. This effect was most pronounced in face and body part selective regions (FFA, OFA, EBA), with the sparsest tuning across all subjects. The face-selective posterior superior temporal sulcus exhibited particularly sparse representation in Subjects 1 and 2, while this region was not present in Subject 3 and, as expected, also yielded no increase in sparseness. Scene-selective regions (PPA, MPA, OPA) also exhibited sparseness, though the effects were more variable across subjects, which could arise from the representational dimensions being derived from objects within scenes, as opposed to isolated scene images without a focus on individual objects. Conversely, non-category-selective regions, such as early visual cortices, clearly exhibited dense representations. These findings suggest that category-selective regions, while responsive to multiple dimensions, may primarily respond to a small subset of behaviorally-relevant dimensions. Thus, in a multidimensional representational framework, category-selectivity may reflect a special case of sparse tuning within a broader set of distributed dimension tuning maps.

Beyond the increased sparseness in functionally selective clusters, which had been defined in an independent localizer experiment ^57^, we explored to what degree we could use sparseness maps for revealing additional, potentially novel functionally selective regions. To this end, we identified two clusters with consistently high sparseness values across subjects (Fig. 5B). One cluster was located in the right hemisphere anterior to anatomically-defined area FG4 ^83^ and between functionally-defined FFA and anterior temporal face patch ^84^, with no preferential response to human faces in 2 of 3 subjects in a separate functional localizer. The other cluster was located in orbitofrontal cortex, coinciding with anatomically defined Fo3 between the olfactory and medial orbital sulci ^85^. Having identified these clusters, we extracted regional tuning profiles and determined the most representative object images for each cluster. Inspection of the tuning profiles in these sparsely tuned regions revealed that their responses were best captured by images of animal faces for the region anterior to FFA and sweet food for orbitofrontal cortex (Fig. 5C). While the results in orbitofrontal cortex are in line with the motivational significance of rewarding foods and food representations in frontal regions ^74,86–89^, the selective response to animal faces in the cluster anterior to FFA deserves further study. By identifying regional response selectivity in a data-driven fashion ^90^, the results show that sparse tuning can aid in localizing functionally selective brain regions, corroborating the link between representational dimensions and regional selectivity.

### Object dimensions offer a better account of visual cortex responses than categories

If representational dimensions offer a better account of the function of ventral visual cortex than categorization, this would predict that they have superior explanatory power for brain responses to visually-perceived objects in these regions ^47,91^. To compare these accounts formally, we compiled a multidimensional and a categorical model of object responses and compared the amount of shared and unique variance explained by these models. We first constructed a category model by assigning all objects appearing in the presented images into 50 common high-level categories (e.g. “animal”, “bird”, “body part”, “clothing”, “food”, “fruit”, “vehicle”) available as part of the THINGS metadata ^92^. Then, for each category, we determined the most diagnostic object dimension. Since some dimensions mapped to multiple categories, this resulted in a model of 30 object dimensions. Note that this approach of using an unequal number of regressors in model comparison biases results in favor of the object category model, thus representing a conservative test of our prediction. Based on the 50 categories and the 30 dimensions, we fit two encoding models to the fMRI single trial responses and performed variance partitioning to disentangle the relative contribution of the object category and dimension models to the cross-validated prediction.

**Fig. 6.**
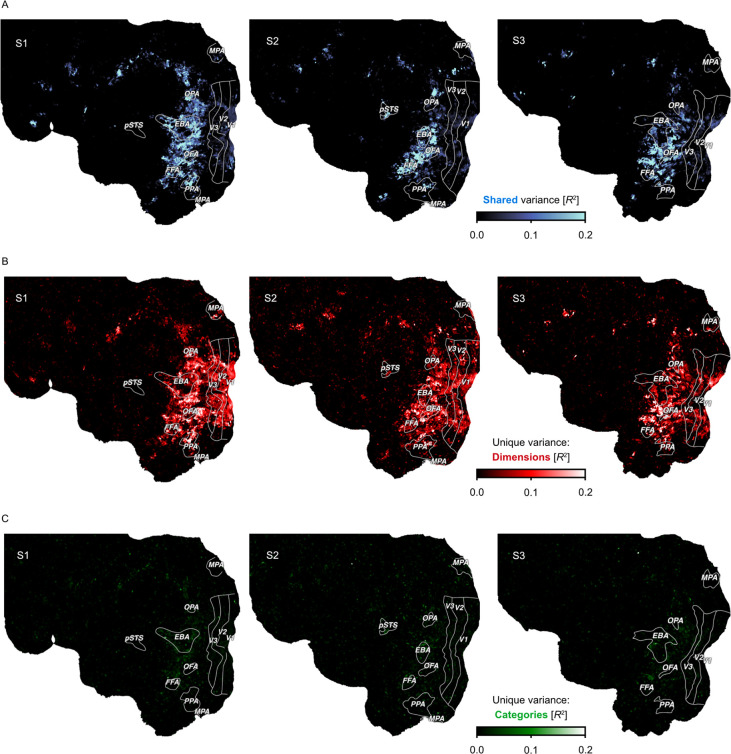
Comparison of a continuous dimensional and a categorical model of object responses. Flat maps show the left hemisphere of each subject. Colors indicate the proportion of explained variance (noise ceiling corrected R^2^) from variance partitioning. A. Shared variance in single-trial fMRI responses explained by both models. B. Variance explained uniquely by a multidimensional model. C. Variance explained uniquely by a model of object categories.

The results (Fig. 6) demonstrate that both object dimensions and categories shared a large degree of variance in explaining brain responses, especially in higher-level ventro-temporal and lateral occipital cortices, suggesting that both models are well suited for predicting responses in the visual system. However, when inspecting the unique variance explained by either model, object dimensions explained a much larger amount of additional variance than object categories. This gain in explained variance was not only evident in higher-level regions, where both models performed well, but extended across large parts of visual cortex, suggesting that behaviorally-relevant dimensions captured information not accounted for by categories. Conversely, category membership added little unique explained variance throughout the visual system. Together, these results indicate that a multidimensional model offers an account with more explanatory value than a category model, supporting the idea that capturing behaviorally-relevant responses in the visual systems requires moving beyond categorization and suggesting object dimensions as a suitable model of encoding the behavioral significance of objects.

## Discussion

Determining how the human brain represents object properties that inform our broad range of behaviors is crucial for understanding how we make sense of our visual world and act on it in meaningful ways. Here, we identified behaviorally-relevant brain representations by predicting fMRI responses to thousands of object images with 66 interpretable representational dimensions underlying millions of object similarity judgements. The results revealed that this behaviorally-relevant information is mirrored in activity patterns throughout the entire visual processing hierarchy in the form of distributed tuning maps, emphasizing the importance of considering the entire system for identifying the behavioral relevance of visual responses. The diverse image selectivity of different visual brain regions emerged from the multidimensional tuning profiles in this distributed representation. This suggests that behaviorally-relevant dimensions offer a broadly applicable model for understanding the architecture of the visual system in which category-selective regions stand out as a special case of sparse tuning. A direct model comparison confirmed that such a multidimensional account has more explanatory value than a category-centric account.

Much work on the behavioral relevance of object responses in occipitotemporal cortex has focused primarily on a limited number of behavioral goals, such as recognition and categorization ^20–22,28,70,91^. According to this view, high-level visual regions contain representations that abstract from factors non-essential for recognition and categorization, such as position, color, or texture ^3,93,94^. Our findings provide an alternative perspective onto the nature of cortical object representations that may offer greater explanatory power than this traditional view. By considering a richer representation of objects supporting broader behavioral goals ^23^, object information is no longer restricted to the commonalities between objects based on how we label them. In this framework, even responses in early visual cortex to images from high-level categories such as food ^73,74^, which would traditionally be disregarded as lower-level confounds based on texture or color, are relevant information supporting the processing of behaviorally-relevant visual inputs. In this perspective, object vision solves the more general problem of providing a rich representation of the visual environment capable of informing a diverse array of behavioral domains ^23^.

While our results favor a distributed view of object representations, localized response selectivity for ecologically important object stimuli has been replicated consistently, underscoring the significance of specialized clusters. Regional specialization and distributed representations have traditionally been seen as opposing levels of description ^37,38^. In contrast, our study advances a framework for unifying these perspectives by demonstrating that, compared to other visual regions, category selective clusters exhibit sparse response tuning profiles. This framework treats regional specialization not as an isolated phenomenon, but rather a special case within a more general organizing principle. Thus, it provides a more general view of object representations that acknowledges the significance of regional specialization in the broader context of a multidimensional topography.

One limitation of our study is that we did not identify behaviorally-relevant dimensions specific to each individual participant tested in the MRI. Instead, dimensions were based on a separate population of participants. However, our findings were highly replicable across the three participants, suggesting that the dimensions we used reflect general organizing principles rather than idiosyncratic effects. Future work could test the extent to which these results generalize to the broader population and how they vary between individuals. Further, despite the broad diversity of objects used in the present study, our work excluded non-object images like text ^66^. While effects of representational sparseness were less pronounced in scene-selective regions and largely absent in text-selective regions ^10^, our encoding model significantly predicted brain responses in scene-selective regions (Suppl. Fig. 2), indicating validity beyond isolated objects. Future research may extend these insights by exploring additional image classes.

While the behaviorally-relevant dimensions used in this study were highly predictive of perceived similarity judgments and object categorization ^52^, there are many possible behaviors not captured by this approach. Here, we used representational dimensions underlying similarity judgments to contrast with the category-centric approach. We chose similarity judgments as a common proxy for mental object representations, since they underlie various behavioral goals, including categorization and recognition ^52–56^. Future work could test the extent to which other behaviors or computational approaches carry additional explanatory value ^15,49,51,95,96^. This would also allow establishing the causal relevance of these activity patterns in behavioral readout ^13,15,17,97^.

Given the explanatory power of our dimensional framework, our results may be interpreted as hinting at an alternative explanation of traditional stimulus-driven feature selectivity through the lens of behavioral relevance ^98^, where the emergence of feature selectivity may exist because of the potential for efficient behavioral readout. Since the dimensions used in this study likely do not capture all behaviorally-relevant selectivity, our approach does not allow testing this strong assumption. For example, a direct comparison of our embedding with the predictive performance of state-of-the-art deep neural network models ^99^ would neither support nor refute this idea. However, future work could specifically target selectivity to individual visual features to determine the degree to which these representations are accessible to behavioral readout and, thus, may alternatively be explained in terms of behavioral relevance, rather than feature selectivity.

In conclusion, our work provides a multidimensional framework that aligns with the rich and diverse behavioral relevance of objects. This approach promises increased explanatory power relative to a category-centric framework and integrates regional specialization within a broader organizing principle, thus offering a promising perspective for understanding how we make sense of our visual world.

## Methods

### THINGS-data

We relied on the openly available THINGS-data collection to investigate the brain representation of every-day objects ^57^. THINGS-data includes 4.7 million human similarity judgements as well as neural responses measured with functional magnetic resonance imaging (fMRI) to thousands of naturalistic and broadly sampled object images. The collection also includes a representational embedding of core object dimensions learned from the similarity judgments, which predicts unseen human similarity judgements with high accuracy and offers an interpretable account of the mental representation of objects ^52,57^. Here, we used these object dimensions to predict fMRI responses to object images. All data generation and processing methods are described in detail in the original data publication ^57^ and are only summarized here.

### Participants

The MRI dataset in the THINGS-data collection comprises data from 3 healthy volunteers (2 female, 1 male, mean age: 25.33 years). Participants had normal or corrected-to-normal visual acuity, and were right-handed. The behavioral dataset in the THINGS-data collection was obtained from 12,340 participants through the crowdsourcing platform Amazon Mechanical Turk (6,619 female, 4,400 male, 56 other, 1,065 not reported; mean age: 36.71, std: 11.87, n=5,170 no age reported).

### Stimuli

All images were taken from the THINGS database ^66^. The THINGS database contains 26,107 high-quality, colored images of 1,854 object concepts from a wide range of nameable living and non-living objects, including non-countable substances (e.g. “grass”), faces (e.g. “baby”, “boy”, “face”), and body parts (e.g. “arm”, “leg”, “shoulder”). The stimuli presented during functional MRI included 720 object concepts from the THINGS database, with the first 12 examples of each concept selected for a total of 8,640 images. In addition, 100 of the remaining THINGS images were presented repeatedly in each session for the purpose of estimating data reliability.

### Experimental procedure

Participants of the THINGS-fMRI experiment took part in 15–16 scanning sessions, with the first 1–2 sessions serving to acquire individual functional localizers for retinotopic visual areas and category-selective clusters (faces, body parts, scenes, words, and objects). The main fMRI experiment comprised 12 sessions where participants were presented with the 11,040 THINGS images (8,740 unique images, catch trials excluded, 500 ms presentation followed by 4 s of fixation). For details on the procedure of the fMRI and behavioral experiments, please consult the original publication of the datasets ^57^.

Behavioral similarity judgements in the THINGS-data collection were collected in a triplet odd-one-out study using the online crowdsourcing platform Amazon Mechanical Turk. Participants were presented with three object images side by side and were asked to indicate which object they perceived to be the odd-one-out. Each task comprised 20 odd-one-out trials, and participants could perform as many tasks as they liked.

### MRI data acquisition and preprocessing

Whole-brain functional MRI images were acquired with 2mm isotropic resolution and a repetition time of 1.5s. The MRI data was preprocessed with the standard pipeline fMRIPrep ^100^ which included slice time correction, head motion correction, susceptibility distortion correction, co-registration between functional and T1-weighted anatomical images, brain tissue segmentation, and cortical surface reconstruction. Additionally, cortical flat maps were manually generated ^101^. Functional MRI data was denoised with a semi-automated procedure based on independent component analysis (ICA) which was developed specifically for the THINGS-fMRI dataset. The retinotopic mapping data and functional localizer data were used to define retinotopic visual regions as well as the category-selective regions used in this study. Image-wise response estimates were obtained by fitting a single-trial model to the fMRI time series of each functional run while accounting for variation in hemodynamic response shape and mitigating overfitting ^102–104^.

### Behavioral embedding

In order to predict the neural response to seen objects, we used a recent, openly available model of representational dimensions underlying human similarity judgements of objects ^52^. This model was trained to estimate a low-dimensional, sparse, and non-negative embedding predictive of individual trial choices in an odd-one-out task on 1,854 object images. The dimensions of this embedding have been demonstrated to be highly predictive of human similarity judgments while yielding human-interpretable dimensions reflecting both perceptual (e.g. “red”, “round”) as well as conceptual (e.g. “animal-related”) object properties. We used a recent 66-dimensional embedding trained on 4.7 million odd-one-out judgments on triplets of 1,854 object images ^57^.

While the original embedding was trained on one example image for each of the 1,854 object concepts, it may not account for differences between exemplars of the same object concept. For example, the color of the apple the model was trained on might have been red, while we also presented participants with images of a green apple. This may underestimate the model’s potential to capture variance in brain responses to visually presented object images. To address this, we extended the original embedding by predicting the 66 object dimensions for each individual image in the THINGS database ^66^. To this end, we used the neural network model CLIP-ViT, which is a multimodal model trained on image-text pairs and which was recently demonstrated to yield excellent prediction of human similarity judgments ^65,105^. For each of the 1,854 object images, we extracted the activity pattern from the final layer of the image encoder. Then, for each of the 66 dimensions, we fitted a ridge regression model to predict dimension values, using cross-validation to determine the regularization hyperparameter. Finally, we applied the learned regression model to activations for all images in the THINGS database. This resulted is a 66-dimensional embedding that captures the mental representation of all 26,107 THINGS images. We used these predicted dimension values to predict fMRI responses to the subset of 8,740 unique images presented in fMRI, which yielded consistent improvements in explained variance for all dimensions (see Supplementary Fig. 1).

### Encoding model

We used a voxel-wise encoding model of the 66-dimensional similarity embedding to predict image-wise fMRI responses in order to test 1) how well the model predicts neural responses in different parts of the visual system and 2) how neural tuning to individual dimensions maps onto the topography of visual cortex.

#### Linear regression on fMRI single trial estimates

To test how well the core object dimensions predict brain responses in different parts of the visual system, we fit them to the fMRI single trial response estimates using ordinary least squares regression. While most analyses in this work rely on a more powerful parametric modulation model estimated on time-series data (see below), we used single trial responses for estimating the predictivity of the object dimensions, since this approach does not require extracting the contribution of the parametric modulators for estimating the explained variance of the general linear model. We evaluated the prediction performance of this encoding model in a leave-one-session-out cross-validation, using the average correlation between predicted and observed fMRI responses across folds. Within each cross-validation fold, we also computed a null distribution of correlation values based on 10,000 random permutations of the held-out test data. To assess statistical significance, we obtained voxel-wise *p*-values by comparing the estimated correlation with the generated null-distribution and corrected for multiple comparisons based on a false discovery rate of *p* < 0.01. For visualization purposes, R^2^ values were normalized by the noise ceiling estimates provided with the fMRI dataset ^57^.

#### Parametric modulation on fMRI time series

In order to evaluate the contribution of individual object dimensions to the neural response in a given voxel, we used a parametric modulation model on the voxel-wise time series data. In this parametric modulation, a general onset regressor accounts for the average response across all trials, and a set of 66 parametric modulators account for the modulation of the BOLD signal by individual object dimensions. To compile the parametric modulation model, we constructed dimension-specific onset regressors and mean-centered each parametric modulator in order to make them orthogonal to the general onset regressor. We then convolved these regressors with a hemodynamic response function (HRF) to obtain predictors of the BOLD response. To account for variation in the shape of the HRF, we determined the best fitting HRF for each voxel based on a library of 20 HRFs ^102,103^. The resulting design matrix was then concatenated and fit to the fMRI time-series data. In order to mitigate overfitting, we regularized the regression weights using fractional ridge regression ^104^. We chose a range of regularization parameters from 0.10 to 0.90 in steps of 0.10 and from 0.90 to 1.00 in steps of 0.01 in order to sample values more densely which reflect less regularization. We determined the best hyperparameter combination (20 HRFs, 26 regularization parameters) for each voxel based on the amount of variance explained in a 12-fold between-session cross-validation. Finally, we fit the model with the best hyperparameter combination per voxel to the entire dataset, yielding 66 statistical maps of regression weights representing the voxel-wise contribution of individual object dimensions in predicting the fMRI signal. The regularization hyperparameter turned out to be small throughout visual cortex (Suppl. Fig. 3), demonstrating that regularization of regression weights had little impact on the absolute size of regression weights.

### Regional tuning profiles and most representative object images

To explore the functional selectivity implied by regional tuning to core object dimensions, we extracted tuning profiles for different visual brain regions and related them to the multidimensional representation of all object images in the THINGS database ^66^ using a high-throughput approach. First, we extracted the regression weights resulting from the parametric modulation model in different visual brain regions: V1, V2, V3, human V4 (hV4), occipital face area (OFA), fusiform face area (FFA), extrastriate body area (EBA), parahippocampal place area (PPA), medial place are (MPA), and occipital place area (OPA). We then averaged these regional tuning profiles across participants and set negative weights to zero, given that the predicted dimensions reflect non-negative values, as well. We plotted the regional tuning profiles as rose plots to visualize the representation of core object dimensions in these brain regions. In order to explore the regional selectivity for specific object images, we determined the cosine similarity between each regional tuning profile and the model representation of all 26,107 images in the THINGS database. This allowed us to identify those THINGS images that are most representative of the local representational profile in different visual brain regions.

### Representational sparseness

We estimated the sparseness of the representation of core object dimensions based on the regression weights from the parametric modulation model. Given our aim of identifying local clusters of similarly-tuned voxels, we performed spatial smoothing on the regression weight maps (FWHM = 4mm) to increase the spatial signal-to-noise ratio. We then took the vectors representing the 66-dimensional tuning profile for each voxel and removed negative vector elements, mirroring the analysis of the regional tuning profiles. We computed the sparseness of the resulting voxel-wise tuning vectors based on a previously introduced sparseness measure which is based on the normalized relationship between the L-1 and L-2 norm of a vector ^106^:

$$s\left(x\right)=\frac{\sqrt{n}-\sum \left|x_{i}\right|/\sqrt{\sum x_{i}^{2}}}{\sqrt{n}-1}$$

Where s indicates the sparseness of the n-dimensional input vector x. A sparseness value of 1 indicates a perfectly sparse representation where all vector elements except one have the same value. In turn, a value of 0 indicates a perfectly dense representation where all elements have identical values. We computed this sparseness measure over the regression weights in each voxel which yielded a sparseness measure as a single value per voxel. To assess their statistical significance, we first identified the distribution of sparseness values in a noise pool of voxels. This noise pool included voxels where the parametric modulation model predicted the fMRI signal poorly in the cross-validation procedure $\left(R^{2}<0.0001\right)$. Since visual inspection of sparseness histograms suggested a log-normal distribution, we log-transformed all sparseness values to convert them to a normal distribution. Finally, we estimated the mean and standard deviation of the sparseness distribution in the noise pool, allowing us to obtain z- and p-values of the sparseness in each voxel.

Based on these results, we explored whether local clusters of representational sparseness are indicative of brain regions with high functional selectivity. To this end, we identified two regional clusters of high sparseness values which were present in all subjects and which had not yet been defined based on the functional localizer experiment (see MRI data preprocessing). Based on visual inspection of the sparseness maps, we defined two regions of interest. The first region of interest was located in the right ventro-temporal cortex, anterior to anatomically-defined area FG4 ^83^ and functionally-defined FFA, but posterior to the anterior temporal face patch ^84^. The second region of interest was located in the orbitofrontal cortex. We probed the functional selectivity of these sparsely tuned regions by extracting regional tuning profiles and determining the most representative object images as described in the previous section.

### Variance partitioning of object category vs. dimension based models

The aim of the variance partitioning was to test whether object dimensions or object categories offer a better model of neural responses to object images. To this end, we compiled a multidimensional and categorical model and compared the respective amount of shared and unique variance explained by these models.

We used 50 superordinate object categories provided in the THINGSplus metadata collection to construct a category encoding model ^92^. These high-level categories comprised: “animal”, “bird”, “body part”, “breakfast food”, “candy”, “clothing”, “clothing accessory”, “condiment”, “construction equipment”, “container”, “dessert”, “drink”, “electronic device”, “farm animal”, “food”, “footwear”, “fruit”, “furniture”, “game”, “garden tool”, “hardware”, “headwear”, “home appliance”, “home decor”, “insect”, “jewelry”, “kitchen appliance”, “kitchen tool”, “lighting”, “mammal”, “medical equipment”, “musical instrument”, “office supply”, “outerwear”, “part of car”, “plant”, “protective clothing”, “safety equipment”, “school supply”, “scientific equipment”, “sea animal”, “seafood”, “sports equipment”, “tool”, “toy”, “vegetable”, “vehicle”, “watercraft”, “weapon” , and “women’s clothing”. To account for cases where images contained multiple objects (e.g. an image of “ring” might also contain a finger), we used the image annotations in the THINGSplus metadata ^92^ and manually matched these annotations to objects in the THINGS database for all images presented in the fMRI experiment. We then compiled an encoding model with 50 binary regressors encoding the superordinate categories of all respective objects.

Next, we compiled a corresponding encoding model of object dimensions. Note that we predicted that this model would outperform the categorical model in explaining variance in neural responses. To conservatively test this prediction, we biased our analysis in favor of the categorical model by selecting fewer dimensions than categories. To this end, for each category we identified the object dimension with the strongest relationship based on the area under the curve metric (AUC). Since some dimensions are diagnostic for multiple categories (e.g. “animal-related” might be the most diagnostic dimension for both “bird” and “insect”), this resulted in a one-to-many mapping between 30 dimensions and 50 categories. The selected dimensions comprised: “Metallic / artificial”, “food-related”, “animal-related”, “textile”, “plant-related”, “house-related / furnishing-related”, “valuable / precious”, “transportation- / movement-related”, “electronics / technology”, “colorful / playful”, “outdoors”, “paper-related / flat”, “hobby-related / game-related / playing-related”, “tools-related / handheld / elongated”, “fluid-related / drink-related”, “water-related”, “weapon-related / war-related / dangerous”, “household-related”, “feminine (stereotypical)”, “body part-related”, “music-related / hearing-related / hobby-related / loud”, “construction-related / craftsmanship-related / housework-related”, “spherical / voluminous”, “flying-related / sky-related”, “bug-related / non-mammalian / disgusting”, “heat-related / fire-related / light-related”, “foot-related / walking-related”, “head-related”, “medicine-related / health-related”, and “sweet / dessert-related”.

In order to compare the predictive potential of these two models, we fitted them to the fMRI single trial responses in a voxel-wise linear regression and performed variance partitioning. In order to estimate the uniquely explained variance, we first orthogonalize the target model and the data with respect to the other model ^107^. This effectively removes the shared variance from both the target model and the data. We then fit the residuals of the target model to the residuals of the data and calculated the coefficient of determination ($R^{2}$) in a 12-fold between-session cross-validation as an estimate of the unique variance explained by the target model. We then estimated the overall variance explained by both models by concatenating the two models, fitting the resulting combined model to the data, and determining the cross-validated $R^{2}$ estimate. Lastly, we computed an estimate of the shared variance explained by the two models by subtracting the uniquely explained variances from the overall explained variance. For visualization purposes, R^2^ values were normalized by the noise ceiling estimates provided with the fMRI dataset ^57^.

## Extended Data

## Supplementary Material

Supplement 1

## Data Availability

The data supporting our analyses were obtained from the publicly available THINGS-fMRI dataset. The fMRI dataset is accessible on OpenNeuro (https://doi.org/10.18112/openneuro.ds004192.v1.0.5) and Figshare (https://doi.org/10.25452/figshare.plus.c.6161151). The object dimensions embedding underlying behavioral similarity judgements which was used to predict the fMRI responses is available at the Open Science Framework repository (https://osf.io/f5rn6/). The higher-level object category labels which were used to construct a categorical model of object responses are part of the THINGSplus metadata and available at the Open Science Framework (https://osf.io/jum2f/).
