# Supplementary material for: Distributed representations of behavior-derived object dimensions in the human visual system
Source: bioRxiv. 2024 Jul 11:2023.08.23.553812. Originally published 2023 Aug 24. Preprint. [Version 4] doi: 10.1101/2023.08.23.553812 (PMC10473665; doi:10.1101/2023.08.23.553812)
Supplement: Supplement 1 [file media-1.pdf]

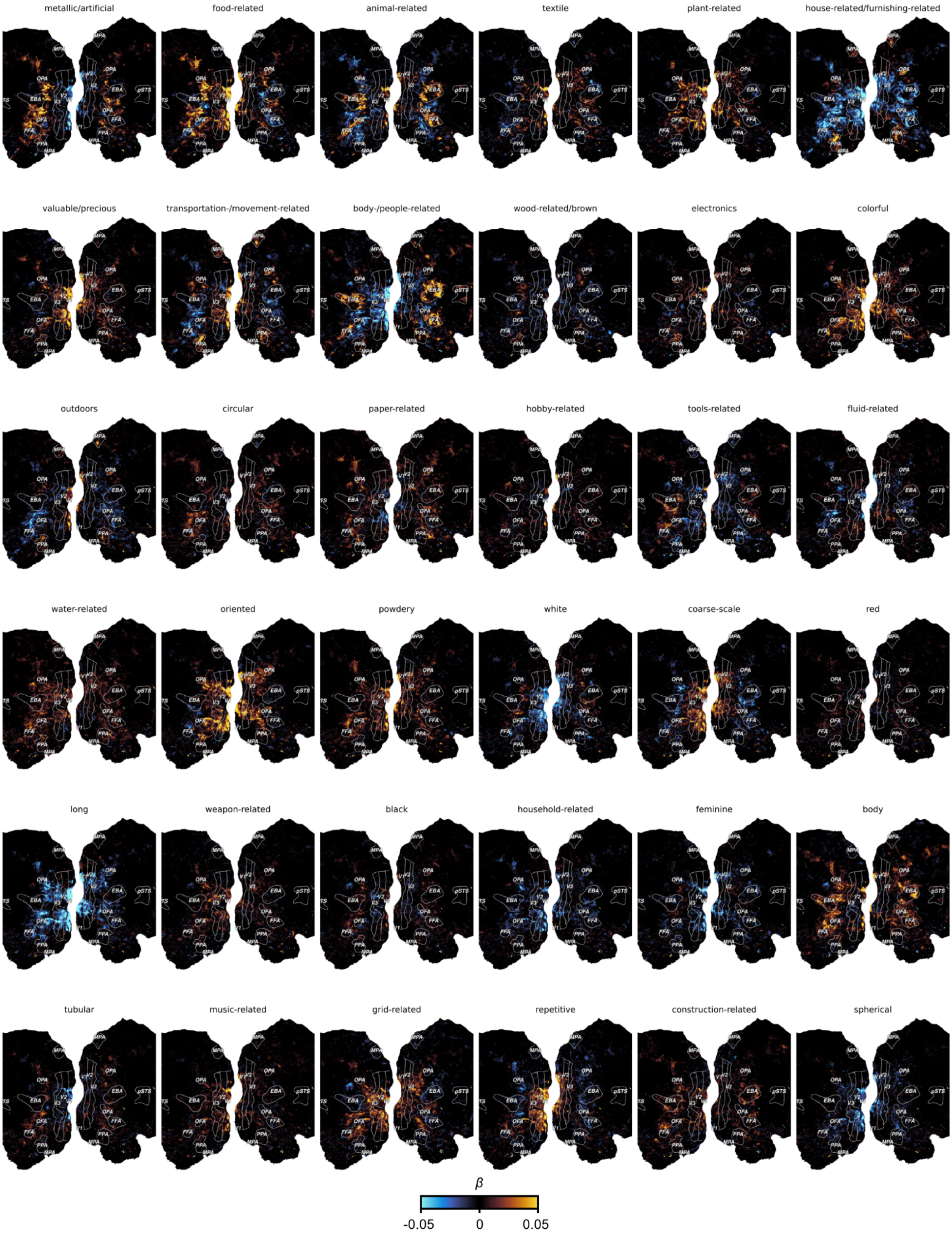

Extended Data Figure 1. **Dimension tuning maps 1-36 for Subject 1**. Colors indicate regression weights for each dimension predictor from the parametric modulation encoding model. Labels indicate regions of interest on the cortex: V1-V3: primary - tertiary visual cortex, OFA: occipital face area, FFA: fusiform face area, EBA: extrastriate body area, PPA: parahippocampal place area, MPA: medial place area, OPA: occipital place area.

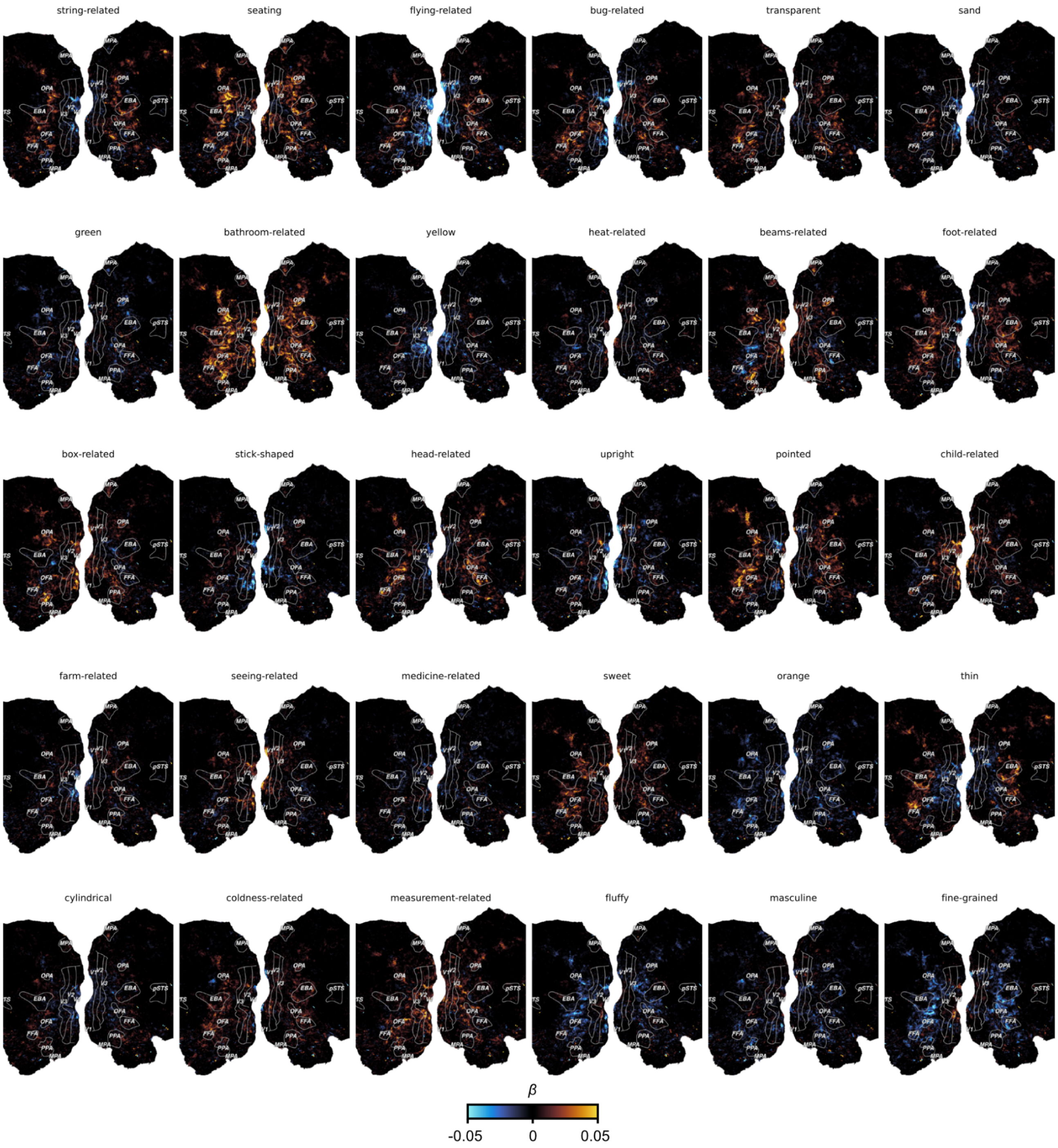

Extended Data Figure 2. **Dimension tuning maps 37-66 for Subject 1**. Colors indicate regression weights for each dimension predictor from the parametric modulation encoding model.

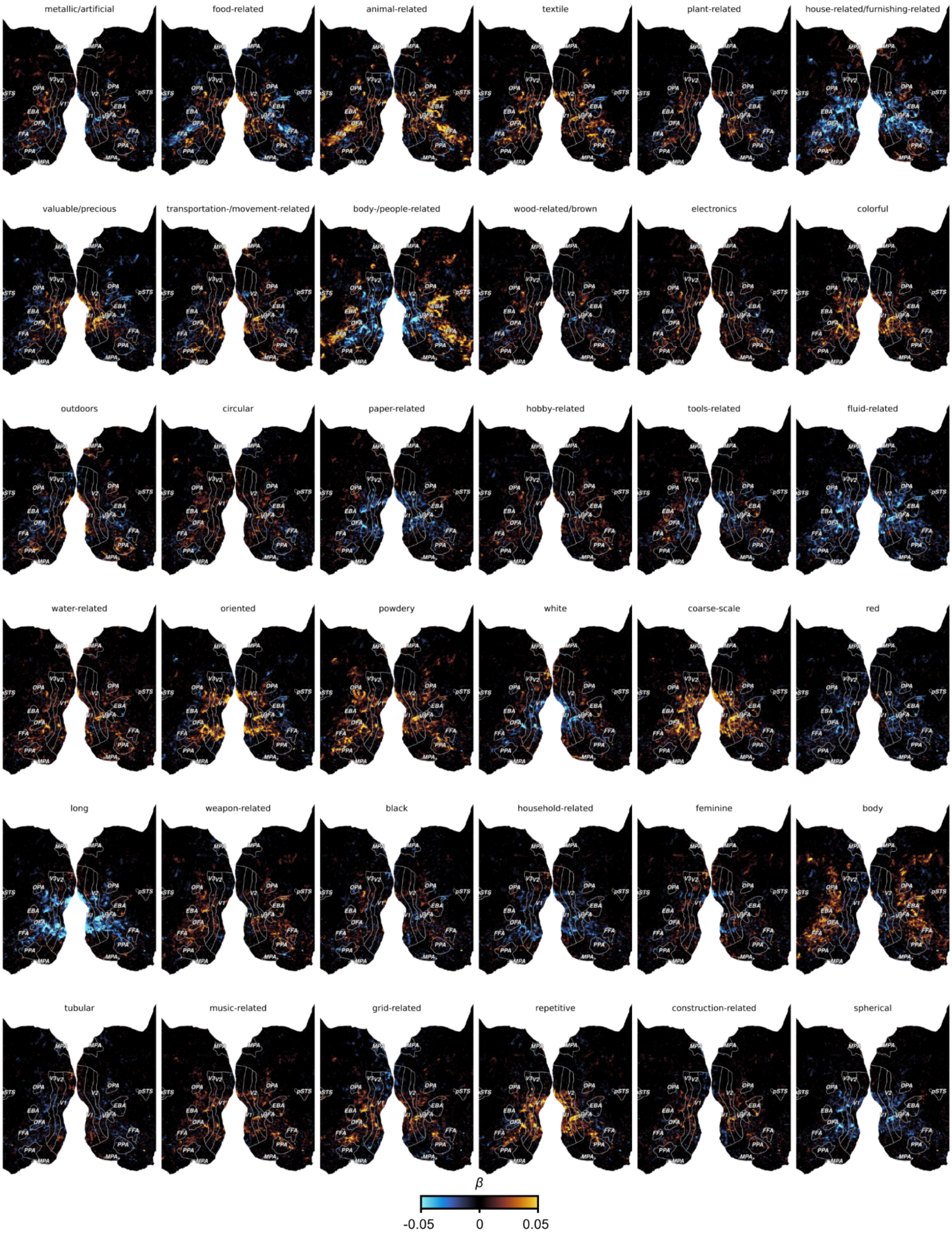

Extended Data Figure 3. **Dimension tuning maps 1-36 for Subject 2**. Colors indicate regression weights for each dimension predictor from the parametric modulation encoding model.

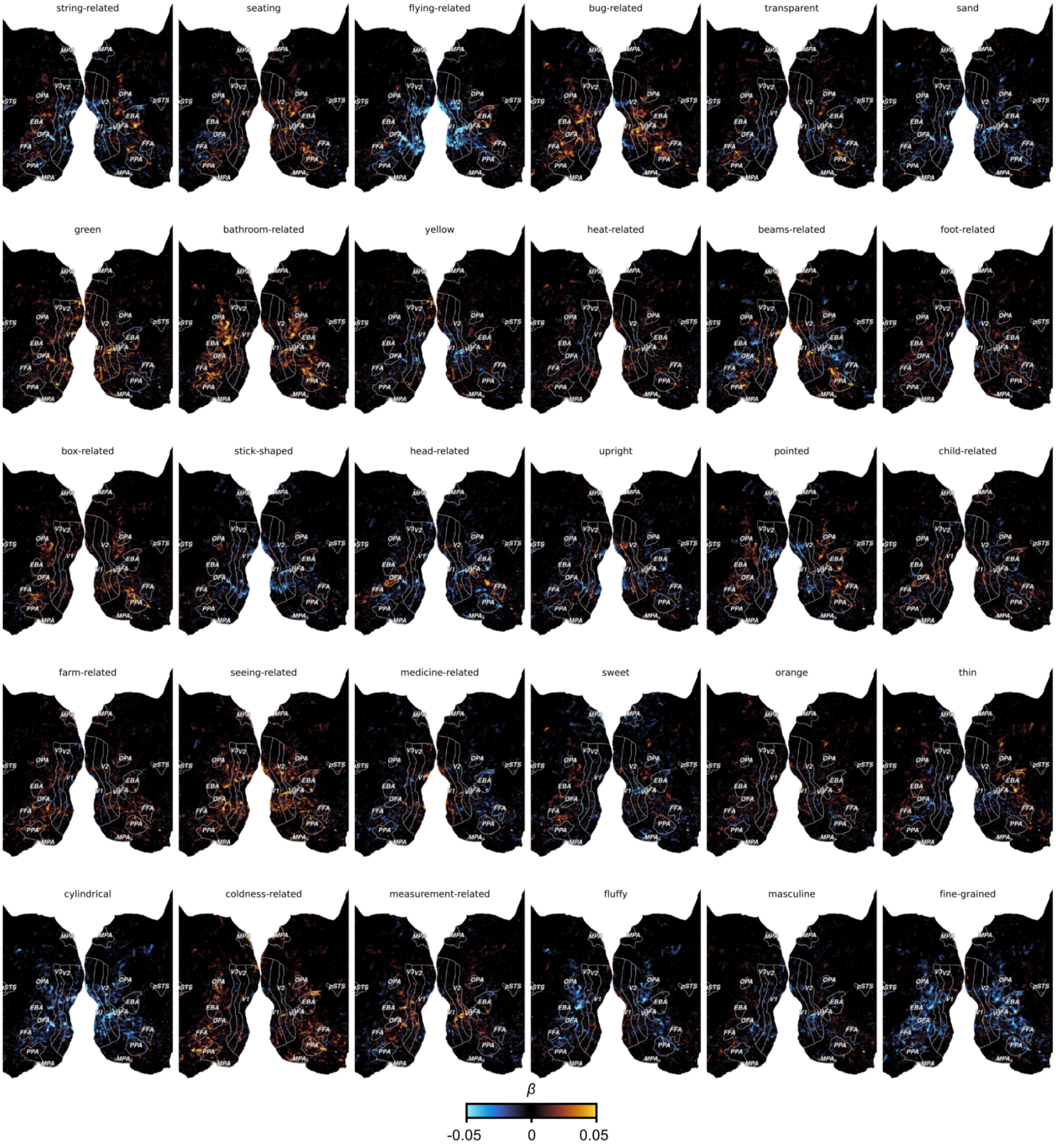

Extended Data Figure 4. **Dimension tuning maps 37-66 for Subject 2**. Colors indicate regression weights for each dimension predictor from the parametric modulation encoding model.

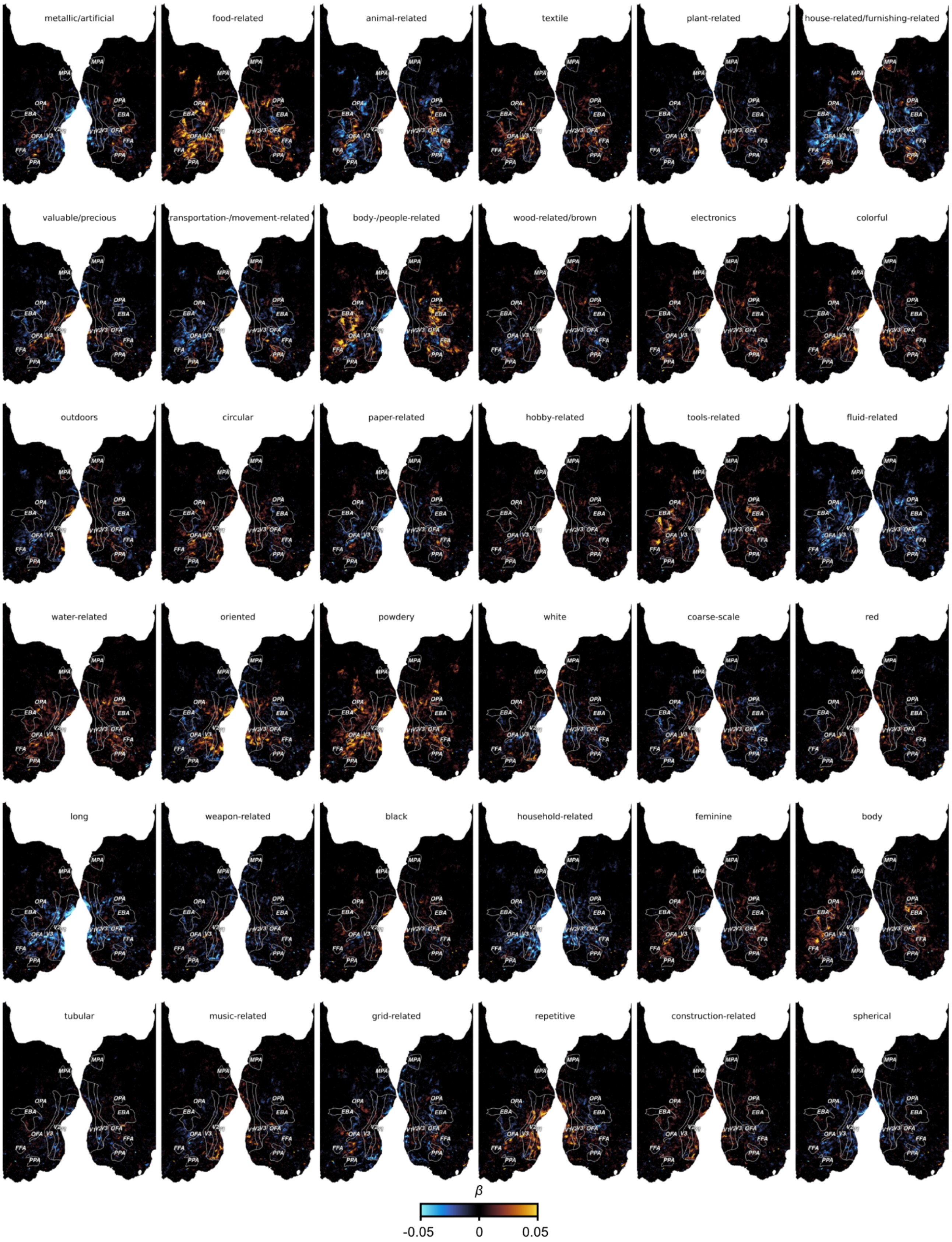

Extended Data Figure 5. **Dimension tuning maps 1-36 for Subject 3**. Colors indicate regression weights for each dimension predictor from the parametric modulation encoding model.

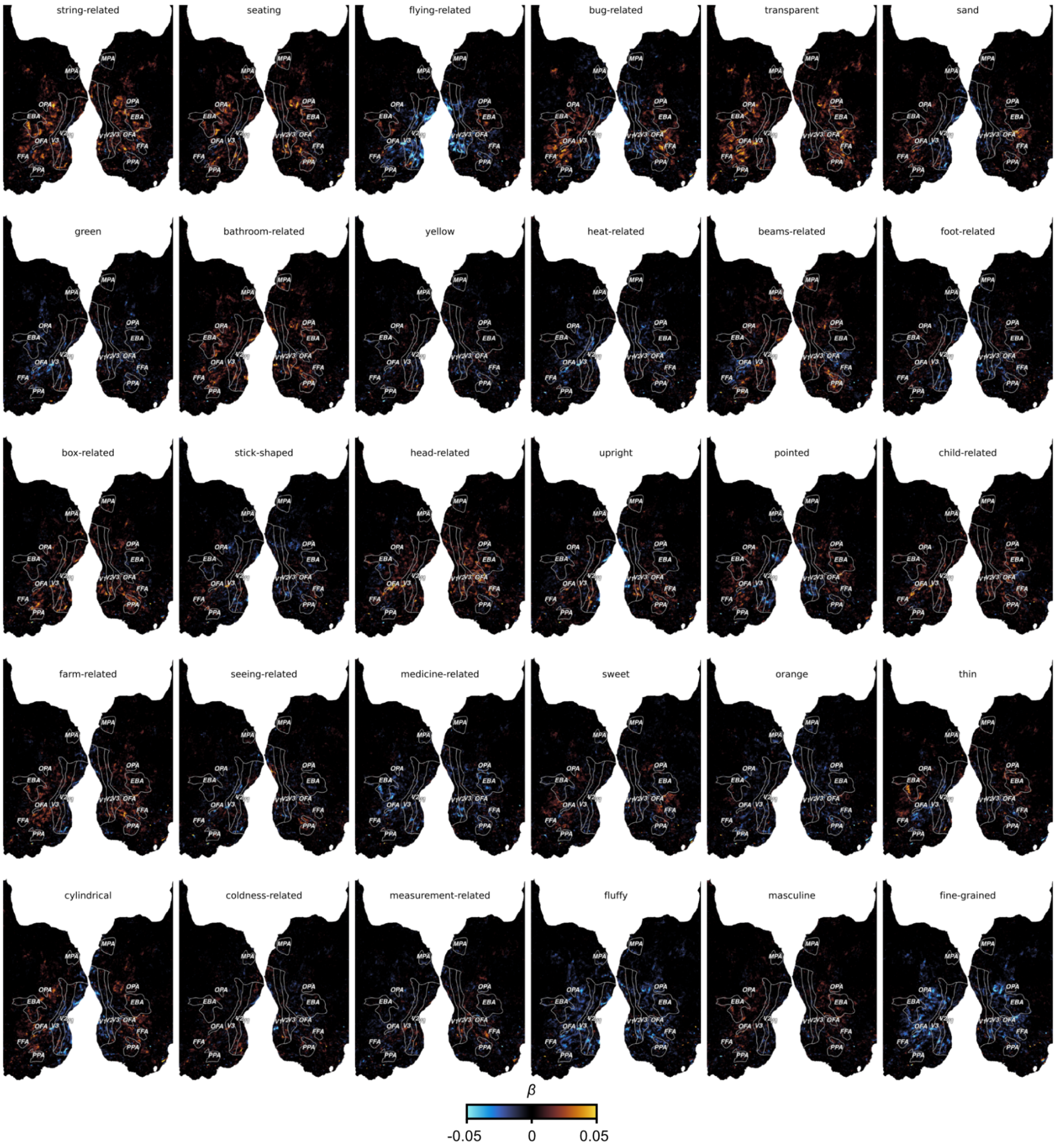

Extended Data Figure 6. **Dimension tuning maps 37-66 for Subject 3**. Colors indicate regression weights for each dimension predictor from the parametric modulation encoding model.
