# Supplementary material for: Distributed representations of behaviorally-relevant object dimensions in the human visual system
Source: bioRxiv. 2023 Nov 20:2023.08.23.553812. Originally published 2023 Aug 24. Preprint. [Version 2] doi: 10.1101/2023.08.23.553812 (PMC10473665; doi:10.1101/2023.08.23.553812)
Supplement: Supplementary file 1 [file NIHPP2023.08.23.553812v2-supplement-1.pdf]

bioRxiv preprint doi: https://doi.org/10.1101/2023.08.23.553812; this version posted November 20, 2023. The copyright holder for this preprint (which was not certified by peer review) is the author/funder. This article is a US Government work. It is not subject to copyright under 17 USC 105 and is also made available for use under a CC0 license.

Supplementary Information

Supplementary Figures

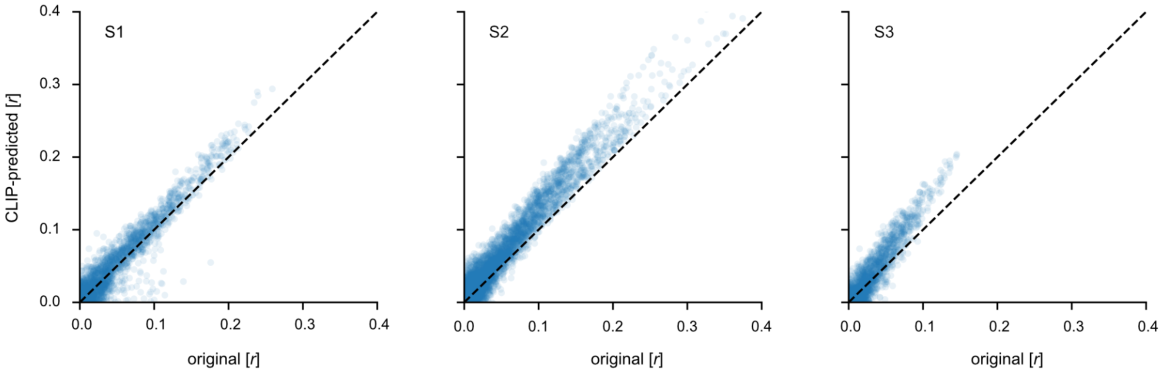

Supplementary Fig. 1. **Improvements in fMRI encoding model accuracy after image-wise prediction of object dimensions**. Scatter plots show the Pearson correlation between held out and predicted data (12-fold cross-validation) for our voxel-wise fMRI encoding model based on the 66 object dimensions underlying perceived similarity [57]. Each sample represents one voxel in a mask of visual cortex (V1-V3, FFA, OFA, pSTS, EBA, PPA, OPA, RSC). The x-axis denotes the prediction performance of the original object embedding based on 1,854 object concepts [57]. The y-axis denotes the prediction performance after these original object dimension weights have been predicted for each individual object image presented in fMRI (see Methods section on behavioral model). The dotted line shows equal performance in both cases.

bioRxiv preprint doi: https://doi.org/10.1101/2023.08.23.553812; this version posted November 20, 2023. The copyright holder for this preprint (which was not certified by peer review) is the author/funder. This article is a US Government work. It is not subject to copyright under 17 USC 105 and is also made available for use under a CC0 license.

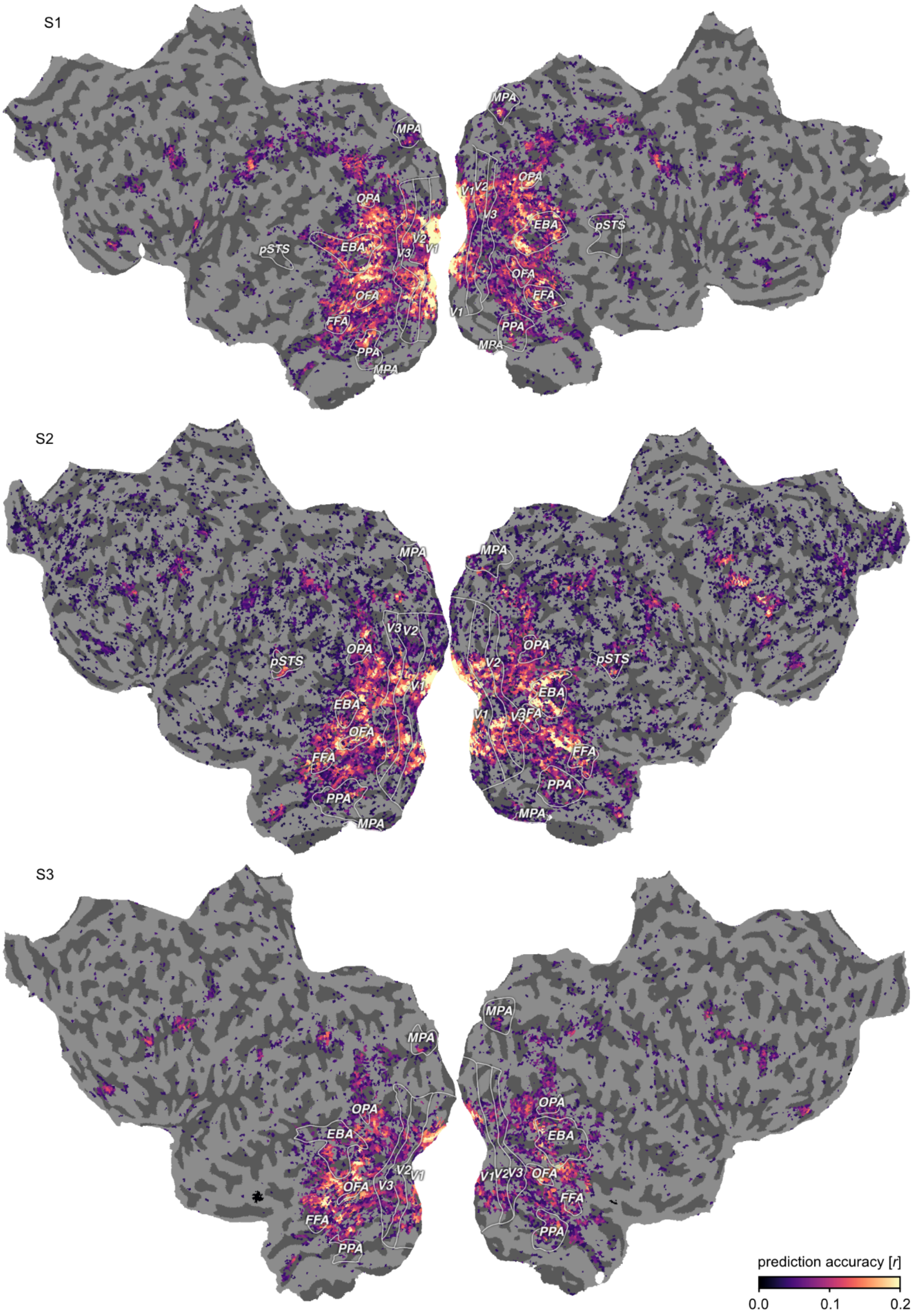

Supplementary Fig. 2. **fMRI encoding model prediction accuracy thresholded for statistical significance**. Each row shows flattened cortical surfaces for one subject. Colors indicate Pearson correlation between predicted and held-out data in a between-session 12-fold cross-validation for all voxels that passed statistical significance ($p<0.01$, FDR-corrected).

bioRxiv preprint doi: https://doi.org/10.1101/2023.08.23.553812; this version posted November 20, 2023. The copyright holder for this preprint (which was not certified by peer review) is the author/funder. This article is a US Government work. It is not subject to copyright under 17 USC 105 and is also made available for use under a CC0 license.

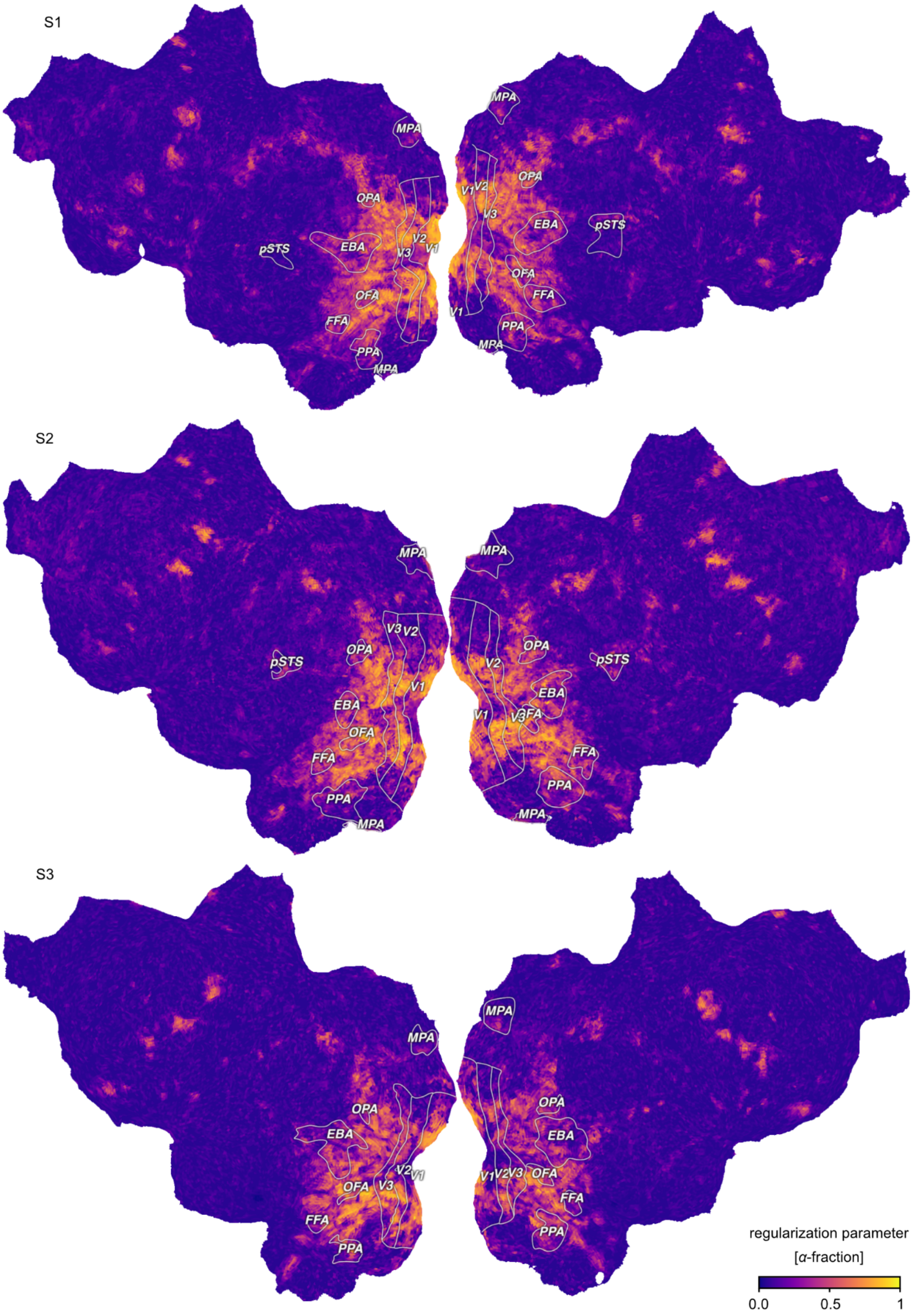

Supplementary Fig. 3. **Regularization parameter in the parametric modulation model**. Colors indicate voxel-wise α-fraction used to regularize the weights in the fractional ridge regression. Larger α-fraction reflect a smaller amount of regularization. An α-fraction of 1 is equivalent to the ordinary least squares solution. An α-fraction of 0 indicates maximum regularization, with all regression weights shrunk to 0.
